# Robot-Assisted Arm Assessments in Spinal Cord Injured Patients: A Consideration of Concept Study

**DOI:** 10.1371/journal.pone.0126948

**Published:** 2015-05-21

**Authors:** Urs Keller, Sabine Schölch, Urs Albisser, Claudia Rudhe, Armin Curt, Robert Riener, Verena Klamroth-Marganska

**Affiliations:** 1 Sensory-Motor Systems Lab, Department of Health Sciences and Technology ETH Zurich, Zurich, Switzerland; 2 Balgrist University Hospital, University of Zurich, Zurich, Switzerland; Rutgers-Robert wood Johnson Medical School, UNITED STATES

## Abstract

Robotic assistance is increasingly used in neurological rehabilitation for enhanced training. Furthermore, therapy robots have the potential for accurate assessment of motor function in order to diagnose the patient status, to measure therapy progress or to feedback the movement performance to the patient and therapist in real time. We investigated whether a set of robot-based assessments that encompasses kinematic, kinetic and timing metrics is applicable, safe, reliable and comparable to clinical metrics for measurement of arm motor function. Twenty-four healthy subjects and five patients after spinal cord injury underwent robot-based assessments using the exoskeleton robot ARMin. Five different tasks were performed with aid of a visual display. Ten kinematic, kinetic and timing assessment parameters were extracted on joint- and end-effector level (active and passive range of motion, cubic reaching volume, movement time, distance-path ratio, precision, smoothness, reaction time, joint torques and joint stiffness). For cubic volume, joint torques and the range of motion for most joints, good inter- and intra-rater reliability were found whereas precision, movement time, distance-path ratio and smoothness showed weak to moderate reliability. A comparison with clinical scores revealed good correlations between robot-based joint torques and the Manual Muscle Test. Reaction time and distance-path ratio showed good correlation with the “Graded and Redefined Assessment of Strength, Sensibility and Prehension” (GRASSP) and the Van Lieshout Test (VLT) for movements towards a predefined position in the center of the frontal plane. In conclusion, the therapy robot ARMin provides a comprehensive set of assessments that are applicable and safe. The first results with spinal cord injured patients and healthy subjects suggest that the measurements are widely reliable and comparable to clinical scales for arm motor function. The methods applied and results can serve as a basis for the future development of end-effector and exoskeleton-based robotic assessments.

## Introduction

Patients who suffer from a neurological disorder such as spinal cord injury (SCI) or stroke often face deficits in motor function. The global-incident rate for traumatic SCI is estimated to be 23 cases per million people (180’000 per year) [1]. Stroke has a prevalence of approximately 795’000 people in the US (Center of Disease Control and Prevention, 2010). These impairments due to stroke or SCI lead to a restriction of both independence and participation in daily life [2, 3]. An intensive rehabilitative intervention can help to improve motor function in stroke [4] and SCI patients [5] and, eventually, the patient’s quality of life.

Plenty of clinical scores and assessments are available for different diseases, ages, movements and body parts to measure patient’s motor functions. The assessments are often categorized using the international classification of functioning, disability and health (ICF) [6] to standardize the description of the health status. With this classification the scores can be grouped according to the disability they address, i.e., body functions and structure, activities and participation. Assessments covering these groups can be used for diagnosis of the patients’ status, as measurement of therapy progress or as feedback about patients’ performance. However, clinical assessments often show deficits in terms of reliability, validity, sensitivity and duration of execution [7].

Rehabilitation robots have the potential to provide an interface for objective, sensitive and reliable measurements. The prevalent use of robots for therapy and the positive findings of robot-assisted therapy contributed to an increased development of robot-assisted assessments in the last five years. Generally two fundamental approaches can be used to evaluate sensorimotor impairment using robot-assisted assessments: Using raw sensor data or feature extraction [7].

The first approach uses **raw sensor data** to directly extract information from sensors about body functions. Depending on the used sensors and parameters, different robot-assisted assessments have already been described. Several approaches focus on assessments of the upper extremity. In time-based assessments the duration is usually measured that is needed to finish a given point-to-point movement or position adjustment of the hand or a joint (e.g. using the MIT Manus [8], the Delta robot [9], the REAplan [10] or the HapticKnob [11]) or by measuring the time needed for a given task (e.g. using the PHANTOM [12] or the MIT Manus [8]). With sensors that measure kinematic or kinetic information, assessments can be performed such as measuring the joint range of motion (ROM) or the workspace (work area) of the hand that can be reached (e.g. using the Lokomat [13], the ACT^3D^ [14], the ArmeoPower [15] or the Microsoft Kinect [16]) or the mean/peak/tangential speed (e.g. using the MIT Manus [8], the MEMOS [17], the IE2000 haptic joystick [18] or the REAplan [10]). An assessment device which can record forces or torques can be used to measure the active joint strength. This can be done recording the maximum voluntary isometric forces or torques (e.g. using the ARMin [19], its commercial version the ArmeoPower [15] or the Lokomat [20]) or isokinetic forces and torques (e.g. using the Kin-Com Dynamometer [21]).

In the **feature extraction** approach the sensor data is further processed, conditioned and characteristic properties are extracted. Using the time and position information during a movement, the quality of the corresponding joint or hand trajectory can be analyzed. Smoothness is a prominent metric to estimate the quality of a movement. Different metrics were used to calculate smoothness such as the ratio between mean speed and peak speed [10, 18, 22, 23], different jerk metrics [10, 11, 17, 22, 23, 24, 25], tent metric [23], mean arrest period [23], peak metric [17, 23, 25], number of submovements [26], comparison with an idealized normal speed profile [27], number of directional changes [22] or the spectral arc length metric [25]. Another feature is the hand-path-ratio [8, 10, 18, 28], sometimes referred to as straightness or trajectory error, which measures the deviation from a given (often straight) trajectory that has to be followed. Moreover, the precision and accuracy of a given targeted movement [17, 27] or the shape accuracy [10, 29] are used as features to describe the quality of a movement. Patients with a neurological disorder often show abnormal synergy [30]. The assessment of abnormal synergies was previously performed using position information [29, 31] or isometric torque values during a motor task [32]. When kinetic information is available in an active device, the resistance to passive movement (RPM) can be extracted as a feature, which can be seen as a measure of spasticity in the measured joint. In this assessment the patient is passive and moved by the isokinetic robot, often at different speeds, while the resistive torque is recorded [33, 34, 35, 36]. Reaction time is a measure for the time the patient needs to initiate a movement. It is usually measured as the time needed between the moving instruction (visual cue, sound) and the movement onset, which is a predefined deviation from the starting position or a velocity threshold [28, 37].

A major challenge is that the robot-assisted assessments are clinically accepted. In clinical environments there already exists a multitude of established clinical assessments and scores that are regularly used and well known by the therapist. A possible approach is, therefore, to reconstruct clinical scores based on the robotic assessment to have an accepted measure understandable for therapists and physicians. Several publications evaluated the correlation between robotic assessments and accepted clinical scores, such as the Fugl-Meyer Assessment ([8], [11], [18], [38]), the Modified Ashworth Scale (MAS) ([8], [11], [13]), the Motor activity log [18], the Action Research Arm Test (ARAT) ([18], [22]), the Jebsen-Taylor Hand Function Test [18], the Graded and Redefined Assessment of Strength, Sensibility and Prehension (GRASSP) [22], Spinal Cord Independence Measure (SCIM) [22], Motor Status Score [8], Motor Power Scale [39], etc.

In this paper we evaluate a set of robot-assisted assessments for the upper extremity in healthy and SCI patients in a stage 1 consideration-of-concept study [40]. Five different assessment packages were implemented that include raw sensor data and feature extraction: ROM (active and passive joint range of motion), WORKSPACE (cubic arm reachable workspace), QOM (quality of arm reaching movements), STRENGTH (isometric joint torques) and RPM (resistance to passive joint movement). The assessments were tested in healthy subjects and SCI patients for applicability, safety, reliability and comparability with clinical scales. The assessments were implemented into the existing therapy robot ARMin III that, through its exoskeleton structure, allows not only for measurement of kinematic- and time-based parameters, but also for kinetic-based measures. The five different assessment packages were combined with a visual interface for an intuitive and standardized execution of the assessments. The implemented assessments are based on measurements used in other devices and are extended and adapted for the use of our robotic setup.

Up to now, most of the robot-assisted arm assessments were tested on stroke patients and the extension of the assessments to the SCI target group was rarely tested. An exception is Zariffa et al [22] who tested different kinematic measures on SCI patients with the passive arm exoskeleton robot ArmeoSpring and Perell et al [33] who investigated muscle tone in SCI patients with an isokinetic dynamometer. However, we are the first to offer a comprehensive measurement of patients’ motor function within one single device.

We hypothesized that the ARMin assessment packages provide an applicable, safe, reliable and comparable tool to measure arm motor functions in SCI patients. In order to evaluate the intra-rater reliability, data from ten healthy subjects was collected. To analyze correlations between clinical tests and ARMin assessments and to quantify the inter-rater reliability, a feasibility study on five SCI patients with two different testers was conducted. We believe that the robotic assessments are widely reliable and comparable to clinical scales for arm motor functions. Furthermore, they may offer a sensitive and objective measurement for more detailed insights in arm motor functions.

## Methods

To evaluate the five ARMin assessment packages—namely ROM, WORKSPACE, QOM, STRENGTH and RPM—healthy subjects and SCI patients participated in this study to investigate the four aspects intra-rater reliability, inter-rater reliability, comparison between healthy subjects and patients, and construct validity.

### ARMin arm rehabilitation robot

**Fig 1:**
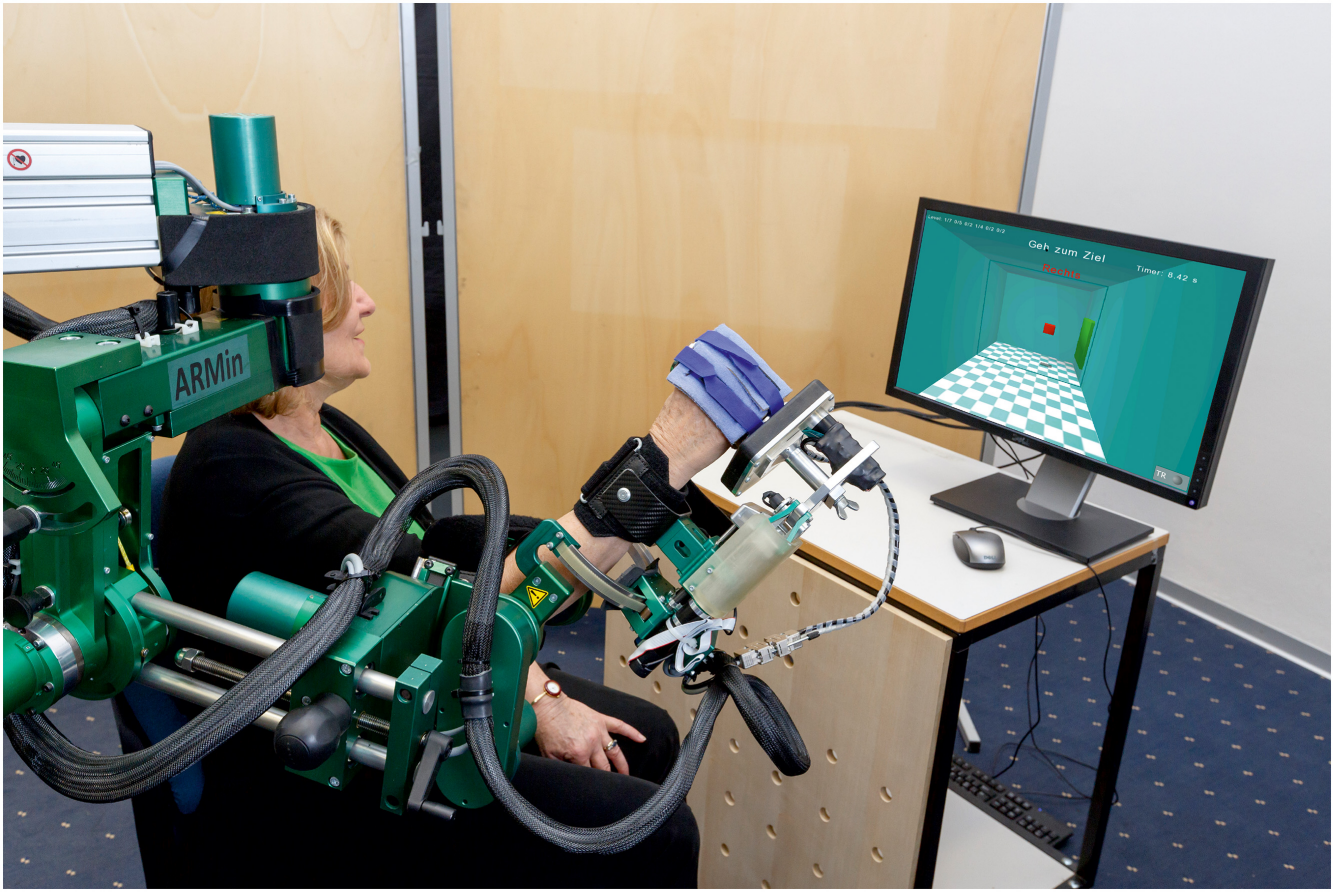
Subject performing assessments with the ARMin arm robot (Courtesy of Dietmar Heinz). Published with written informed consent of the individual in the picture.

The arm therapy robot ARMin has been designed and evaluated by the groups of Riener and Dietz/Curt of ETH Zurich and University of Zurich [41, 42]. ARMin is used for neurorehabilitation of the arm and is characterized by an exoskeleton structure (Fig 1). The latest prototype has seven degrees of freedom allowing 3D shoulder rotation, elbow flexion/extension, pro/supination of the lower arm and wrist flexion/extension. A hand actuation module supports opening and closing of the hand. The patient is sitting on a chair and is fixed in the ARMin robot by the use of cuffs around the upper arm, forearm and around the fingers. The robot can be adjusted to the patient by changing the exoskeleton length settings for the upper arm, the forearm and the hand as well as the shoulder height. The same robot can be used for the training of the left and right arm by changing the hardware configuration. Mechanical end limits are provided for safety reasons to not overstretch joints or collide with the patient. ARMin is the research version of the ArmeoPower (Hocoma AG, Switzerland) and the kinematics, joint ranges and actuation are comparable. The ARMin robot features different control modes covering the range from a tracking only setting, where the robot’s weight and friction are compensated, to complete guidance of the arm. In between, a path control approach can be used to assist the patient as needed. While the path controller is mainly used for the ADL training, the position controller is either used to move the passive patient arm during the RPM assessment or to fix the arm posture during single joint measurements such as ROM or STRENGTH assessment. The compensation mode is used for QOM and WORKSPACE assessments, where the robot should not interfere with the patient’s movement and only follow the arm to record kinematic data.

With its exoskeletal structure, precise position sensors, mechanical transparency and a visual display, ARMin is particularly useful to assess kinetic and kinematic arm functions on joint and end-effector level.

### Participants

Patients’ were recruited by contacting in- and out-patients of the University Hospital of Balgrist. This hospital located in Zurich, Switzerland, offers specialized treatment for SCI patients and treats about 230 inpatients and about 1’500 outpatients after SCI annually.

Eligibility criteria were i) cervical, complete or incomplete SCI with tetraparesis, ii) no severe subluxation of the shoulder, iii) no severe shoulder pain on the tested side and iv) no other illness or incapability that could compromise the assessments. Five patients (aged 19 to 49, mean 33.8, SD 13.8; 4 male) were eligible to perform the five robotic assessments as well as clinical tests. Three patients were in the subacute (i.e. < = 6 months) and two in the chronic (i.e. > 6 months) state post-SCI with different levels of severity (ASIA B to ASIA D) and arm dominance (four right-handed and one left handed) (Table 1). One out of the five patients reported severe shoulder pain on one side, so the corresponding arm was excluded. As the SCI was incomplete in the five patients and their two arms were affected to different degrees, nine unrelated data sets were sampled for evaluation.

**Table 1:** Characteristics of the patients.

Patient ID	Status^a^	Level of lesion	Age in years	Sex	Sequence of arm side tested(r = right, l = left)	Hand dominance after accident^b^(r = right, l = left)	Sequence of tester
1	subacute	sub C5, ASIA B	20	m	r, l	r	T1, T2
2	subacute	sub C5, ASIA B	19	m	l, r	r	T2, T1
3	chronic	sub C4, ASIA C	36	f	r, l	l	T1, T2
4	chronic	sub C6, ASIA B	47	m	r, l	r	T2, T1
5	subacute	sub C1, ASIA D	47	m	l	r	T1, T2

^a^Chronic (> 6 months) vs. subacute (≤ 6 months post SCI).

^b^Evaluated with questionnaire of Chapman [43].

Eleven healthy right-handed subjects (aged 21 to 64, mean 35.4, SD 15.4; 6 male) were assessed on their dominant side to determine the intra-rater reliability. Thirteen healthy right-handed subjects (aged 20 to 68 years, mean 34.9, SD 15.7; 6 male) performed the assessments with their non-dominant arm to compare the performance with the patient’s non-dominant arm. This data was also used to define norm values. None of the subjects had experience with ARMin prior to the study onset.

### Ethics statement

All patients signed an informed consent; the study was approved by the responsible ethical committee (KEK, Zurich, Switzerland) and Swissmedic (Swiss Agency for Therapeutic Products, reference number: 2011-MD-0002). The individual in Fig 1 has given written informed consent (as outlined in PLOS consent form) to publish these case details.

### Study design

In this consideration-of-concept study (stage 1) we aimed to analyze the utility of the chosen assessment measurements and to evaluate how well they can be applied [40]. We tested for applicability, safety, reliability and validity. Forty minutes were scheduled for the performance of the assessment packages, including positing and instruction of the subject.

#### Intra-rater reliability with healthy subjects

The intra-rater reliability was tested with eleven right-handed healthy subjects. A set of norm values was determined. The subjects performed all ARMin assessments four times with their dominant right arm at intervals of one week. Hand dominance was evaluated with the questionnaire of Chapman [43]. No pretest was performed before starting the intra-rater measurement as only minor learning effects were expected. The settings of the robot (arm length settings, shoulder and chair height and position) and the sequence of the ARMin assessments were held constant. Three trained testers performed the measurements, where each subject was always assessed by the same tester.

#### Comparison between healthy subjects and patients

In order to complete a first set of norm values with data from non-dominant arms, 13 healthy subjects performed the assessments once with the non-dominant left hand. Again, three different testers performed the assessments. The acquired norm values for the non-dominant and the dominant arm (values from the first assessment session) were used to compare the assessment performance between the healthy subjects and the SCI patients.

#### Inter-rater reliability and construct validity with patients

For evaluation, inter-rater reliability and correlation between ARMin assessments and common clinical assessments were tested in a cross-sectional study. To assess the inter-rater reliability of the ARMin assessments, two testers performed the assessments. Both trained the ARMin assessments procedure at least ten times and with at least two patients to gain experience with the robot. Testers was given a manual that described the handling of the robot and the instructions to be given to the participants. The order of testers and the order of measured sides of each subject were randomized by lots which the patient drew on day 1. The sequence of ARMin assessments and clinical tests was held constant.

Each patient participated in six sessions (one pretest session, four robotic assessment sessions, one clinical assessments session) that were arranged over a period of twelve days (Table 2). While robotic assessments were conducted by both testers (T1 and T2), all clinical assessments were performed by the same one tester for all patients.

**Table 2:** Study protocol for the patients.

Day 1	Day 2	Day 3	Day 4	Day 5	Day 6	Day 7, Day 8 or Day 9	Day 10	Day 11	Day 12
Pretest	-	-	ARMin test 1	ARMin test 2	-	Clinical tests	-	ARMin test 3	ARMin test 4
robot settings, first test, define tester and arm by lot	-	-	arm 1, T1	arm 1, T2	-	aROM, pROM, MAS, MTS, GRASSP, SCIM, VLT, MMT	-	arm 2, T1	arm 2, T2

### Outcome measures

#### Robotic assessments

Five assessment packages were implemented to evaluate various aspects of arm motor function. While the ROM and the STRENGTH assessment were purely based on raw sensor data, the WORKSPACE, QOM and RPM assessments calculated features that are extracted from the raw data (Table 3). For the QOM and the WORKSPACE assessment the robot is in compensation mode. While most of the gravitational and frictional effects are compensated there may still be forces disturbing the arm movement such as static friction or dynamical effects of the robot.

**Table 3:** Overview over implemented assessments and the measured parameters.

Assessment name	Assessment description	Parameters
ROM	Active and passive ranges of motion of seven joint movements	Active ROM [°] (*aROM*); Passive ROM [°] (*pROM*)
WORKSPACE	Actively achievable Cartesian workspace	*Workspace levels* [# reached levels]/ *Cubic volume* [dm^3^]
QOM	Quality of hand movement while performing goal-directed reaching tasks	Distance-path ratio (*D-P ratio to target*) []; Distance-path ratio (*D-P ratio to start*) []; *Time to target* [ms]; *Time to start* [ms]; *Precision* (Deviation on target) [m]; *Number of peaks to target* []; *Number of peaks to start* []; *Reaction time to target* [ms]; *Reaction time to start* [ms]
STRENGTH	Isometric maximum torque for seven joints	*Joint torques* [Nm]
RPM	Resistance to passive movements for two different speeds in all seven joints	*Joint stiffness* [Nm/rad]

ROM (range of motion) assessment measured active and passive ranges of motion (*aROM* and *pROM*) of the arm. During this assessment ARMin held the patient’s arm in a predefined posture, while the assessed joint was free to move. The patient (*aROM*) or the therapist (*pROM*) moved the free joint in both directions (e.g. flexion and extension), while the software recorded both achievable extreme positions. The order of joints measured was fixed. The predefined robotic postures depended on the joint measured and were chosen to be as similar as possible to the “standardized neutral-0-method positions” [44] (postures described in S1 Table). Seven different joint movements were assessed: shoulder flexion/extension, lateral shoulder ab-/adduction, horizontal shoulder ab-/adduction, shoulder external/internal rotation, elbow flexion/extension, forearm supination/pronation and wrist flexion/extension.

**Fig 2:**
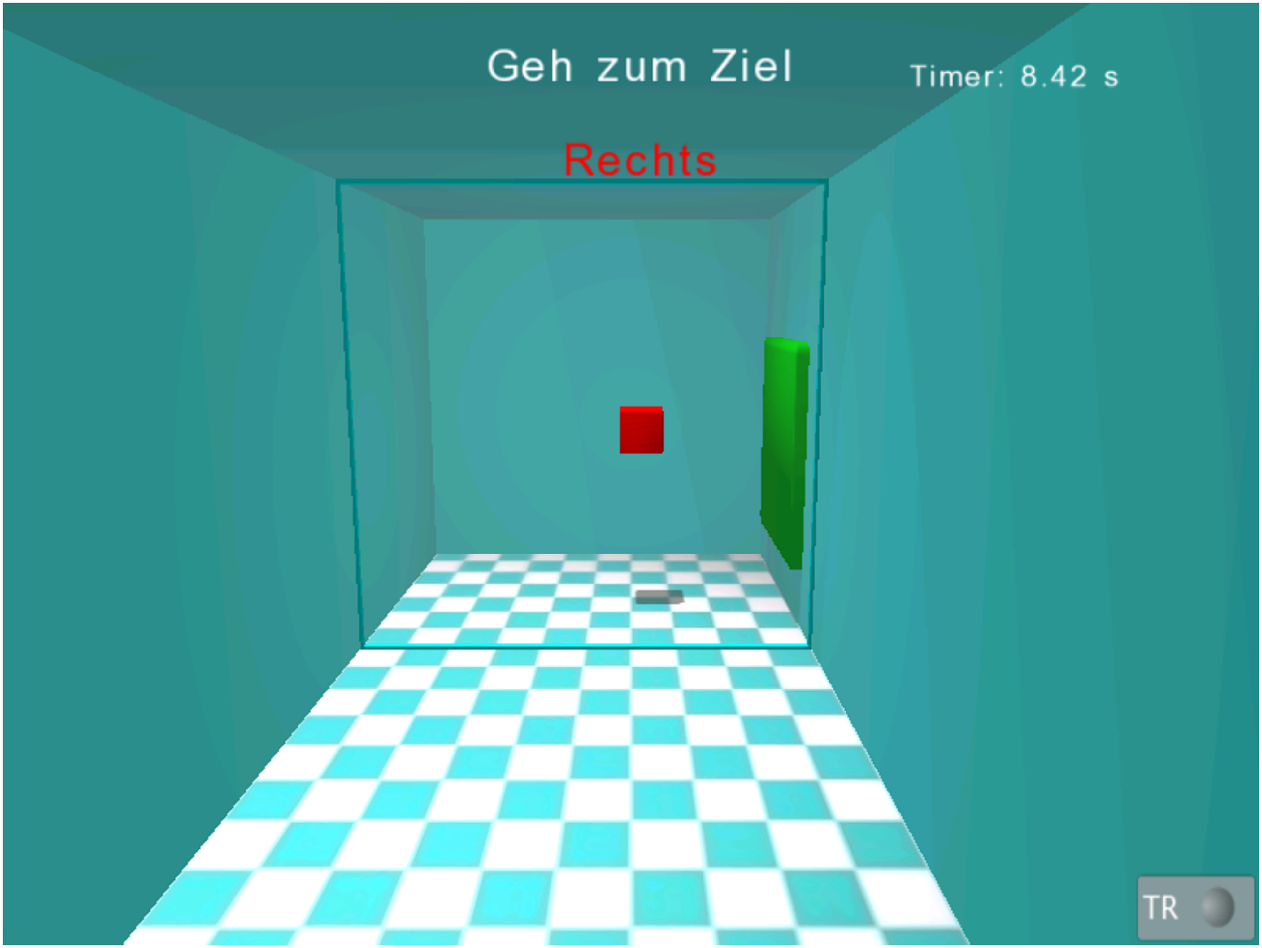
Visual representation of the WORKSPACE assessment package. Screenshot of the WORKSPACE assessment. A room was presented on the screen. The patient looked directly into this room. The end effector of the robot (the position of the patient’s hand) was represented as a small red cube. In the shown situation the patient had to move to the green target position to the right.

WORKSPACE assessment aimed to measure the reachable cubic workspace of the end effector (i.e., the hand). The starting position of the hand was 30 cm in front of the breast (i.e., the xiphoid process of sternum). On the screen, a small cubic room (corresponding to an initial size of 20 cm x 20 cm x 20 cm in the real world) was presented (Fig 2). Each wall in the room indicated a direction to move to (top, bottom, left, right, towards the body, away from the body). For simplification, more directions such as diagonals were not assessed. The aimed movement direction was indicated by a green cube on the wall in randomized order. After the patient had reached the indicated wall, the room grew 5 cm in this direction. If the subject missed an indicated wall the room did not grow in this direction and the direction was shown once more later. The number of discrete increases in a certain direction refers to as a level and was used as an outcome parameter (*workspace level*, in numbers). Furthermore, the achieved room size (*cubic volume*, in dm^3^) was calculated from the workspace levels reached. The maximal volume of the room was 140 dm^3^, the initial size was 8 dm^3^ and the maximal distances to the given room walls were 35 cm for the left and right (five movements in each direction to reach maximum expansion), 30 cm for the top (four movements to reach maximum expansion), and 20 cm for the bottom direction, towards the body and away from the body (two movements in each direction to reach maximum expansion). This results in a total of 20 movements to discretely increase the room size to its maximum.

**QOM (Quality of movement) assessment** measured accuracy and smoothness of a point-to-point movement. Eight target positions appeared successively around a starting position in the frontal plane. The starting position was a circle that allowed some end-effector position variations in the range of 50 mm (rated as “on the start position”). The location of the target positions depended on the volume reached in WORKSPACE, i.e., the targets were at 80% of the reachable distance. The patient was asked to move directly from the start to the target position as soon as it appeared, to rest on the target position until it disappeared (after 3 seconds) and then move directly back to the starting position. Output parameters were the distance-path ratio on the way to the target (*D-P ratio to target*), the standard deviation on the target position (*precision*), and the D-P ratio back to start (*D-P ratio to start*). Furthermore, the *reaction time* to initiate a movement (i.e., the time to leave a circle that was chosen 20% wider than the starting position) and the *time to target* were calculated for each movement. For each movement the *number of peaks* of the end-effector speed (i.e., the zero transition of the end-effector acceleration) was counted as a measure of smoothness (using a peak detection threshold of 7.5% of the maximum speed).

STRENGTH assessment measured the maximum isometric torque of the arm (*joint torques*). ARMin moved the patient’s arm to a predefined measuring posture similar to the one in ROM, but with the measured joint in the midrange (postures are described in S2 Table). The posture was fixed during the joint torque measurement. For a baseline measurement the patient stayed passive for 5 s and then applied the maximum possible torque in the measured joint for 5 s. A simple visual display showed the joint of interest and the timing. This procedure was repeated in both directions of each joint. The applied torque was estimated from the motor current. A moving average filter (window size 1 s) was applied to reduce the effect of single force peaks and the maximal *joint torque* was recorded.

RPM **(Resistance to passive movement) assessment** quantified resistance of a single joint to passive movement within the prior measured *pROM*. ARMin moved the arm to a predefined start posture (identical to the postures in STRENGTH except for the measured joint which moves in the *pROM*, S2 Table). The patient was instructed to keep the arm relaxed and passive while ARMin moved the joint of interest with two different speeds (30°/s, 60°/s). A calibration routine was used to identify the torque contributions that are caused by the robot arm mass. This routine performed the same movements as the RPM assessment but without the patient. A 5^th^ order Butterworth filter with 3 Hz cutoff was used to filter the calculated interaction torques. Knowing the torques applied by the patient the characteristic angle-torque relation could be used to calculate the resistance during the movement. It was assumed that this resistance is a combination of a constant offset torque and a stiffness contribution that changes when the joint angle increases or decreases. Therefore, a linear function was fitted into the data using the least squares method. Further analyzed for this paper was the *joint stiffness* using the slope of the linear fit.

#### Clinical assessments

To evaluate the validity of the robot-assisted assessments the results were compared to clinical outcomes. The active and passive ranges of motion were measured in a sitting position with a handheld goniometer and according to the neutral-0-method [44]. For better comparison with the measurement of WORKSPACE, the arm reachable workspace (ARW) was calculated with a dedicated program as proposed by Klopčar et al [45]. The ARW is based on the subject’s body height, the maximal flexion/extension, abduction/adduction and internal/external rotation of the shoulder and the elbow flexion angle in standing position.

The modified Ashworth Scale (MAS) [46] and the modified Tardieu Scale (MTS) [47] rate the degree of spasticity and were measured for all relevant joints. For the MAS the joint is passively moved with a moderate velocity, for the MTS with high velocity. The tests use an ordinal scale in the range of 0 to 5 for the MAS and 0 to 4 for the MTS, where 0 is equivalent to “no spasticity”.

The GRASSP [48] is an upper extremity assessment for patients after SCI and combines a muscle test for relevant upper extremity muscles, a Semmes-Weinstein-monofilament-test [49] and qualitative and quantitative grasping tasks. In addition to the muscle tests performed within the GRASSP, the Manual Muscle Test (MMT) [50] was performed on muscles that were not covered by the GRASSP but assessed with ARMin. The MMT was conducted in a sitting position. Arm positions were chosen according to the instructions of Daniels and Worthingham [51] and if necessary adapted to the sitting position.

Furthermore, the SCIM [52], a questionnaire for SCI patients that measures the independence in activities of daily living, was conducted to characterize the patients.

To measure functionality of the arm, the scientific or short version of the Van Lieshout Test (VLT) [53] and specific optional items for proximal function of the arm from the clinical or longer version of the VLT (wheelchair propulsion, transfers, push-ups while seated, stabilization of the arms, reaching low, reaching high) were conducted.

### Statistical analysis

The feature extraction for the robotic assessments was performed using MATLAB (Mathworks, R2010b). The resulting parameters were exported to IBM SPSS Statistics for further statistical analysis. All the robotic measures were tested for normal distribution with histograms and Q-Q-Plots. The statistical methods used are summarized in (Table 4).

**Table 4:** Statistical methods for the analysis of the intra-rater reliability, inter-rater reliability and construct validity.

Analyzed aspect	Analyzed data		Statistical method
Intra-rater reliability	Parametric and nonparametric data		Friedman test with pairwise multiple comparison
Inter-rater reliability	Parametric data		ICC (two-way mixed model, single measure)
	Nonparametric data		Spearman’s rank correlation coefficient
	Parametric and nonparametric data		Bland-Altman plot with one-sample Student’s t-test
Construct validity	ROM	manual ROM	Spearman’s rank correlation coefficient, Bland-Altman plot
	WORKSPACE	ARW, VLT, GRASSP, SCIM	Spearman’s rank correlation coefficient
	QOM	VLT, GRASSP, SCIM	Spearman’s rank correlation coefficient
	STRENGTH	MMT	Spearman’s rank correlation coefficient
	RPM	MAS, MTS	Spearman’s rank correlation coefficient

#### Intra-rater reliability

Results of the four repeated assessments in healthy subjects were analyzed with the Friedman test against the null hypothesis that there are no differences. A significant difference (p<0.05) indicates a poor intra-rater reliability. Pairwise multiple comparisons were done by post hoc analysis with Wilcoxon signed-rank tests to determine where the differences between the repeated assessment measures occurred. To analyze the amount of variability between the four measurements the mean difference between the maximum and the minimum value measured was calculated for the aROM, pROM, WORKSPACE, STRENGTH and RPM assessments. For QOM the standard deviation was calculated.

#### Comparison between healthy subjects and patients

To analyze the differences between healthy subjects and patients, a qualitative approach was used calculating the ratio between mean values of the patients and mean values of healthy subjects. The hypothesis was that patients would show a lower performance compared to healthy subjects. Accordingly, for the assessments that produce ascending scores for better performance, the ratio was expected to be lower than 1 in patients. The dominant and non-dominant arms were analyzed independently. For the ROM, rather than comparing the minimal and maximal values of the joint range (e.g., -5° and 130°) the full ranges (for this example, 135°) were calculated and compared to healthy subjects.

#### Inter-rater reliability

For evaluation of the inter-rater reliability nine patient arms were measured. The Spearman’s rank correlation coefficient was calculated for those ARMin assessments which were nonparametric. For parametric data the intraclass correlation coefficient (ICC) was used (two-way mixed model, single measure). A significant correlation in either of these tests indicates a good inter-rater reliability for the assessment.

The differences between the two testers were further analyzed with Bland-Altman plots. The difference of two measurements was plotted against the mean of the two measurements. The one-sample Student’s t-test was used with the null hypothesis that the mean difference between the testers is zero. A significant Student’s t-test (p <0.05) indicates a significant difference between results of testers 1 and 2. The Bland-Altman plots were calculated without outliers (rejected by visual inspection; number of outliers: ROM: 20/252, WORKSPACE: 0/63, QOM: 31/514, STRENGTH: 7/128, RPM: 4/184).

#### Construct validity

The construct validity was analyzed by comparing ARMin assessment parameters of the patient arms with clinical measurements, using the Spearman’s rank correlation coefficient. The ARMin assessments and corresponding clinical tests are listed in Table 4. Mean values of the ARMin assessment parameters of testers 1 and 2 were used. Values which were rated as outliers by visual inspection in the reliability analysis were excluded from validity analysis as well. A correlation of 0.0–0.5 was considered as weak, 0.5–0.75 as moderate and 0.75–1.0 as strong correlation. Correlations with a significance level p<0.05 are marked with *, correlations with significance level <0.01 with ** in the text.

## Results

Only STRENGTH values were normally distributed. Nonparametric methods were used for analysis in the other assessments.

### Intra-rater reliability

Intra-rater reliability was calculated from four complete assessment sessions performed in eleven healthy subjects. The results are summarized in Table 5. The amount of variability between the four tests can be seen in S4 Table.

**Table 5:** Summary of the significant differences found from the Friedman test of the intra-rater reliability.

Assessment	Significant differences between the parameters from the four assessment tests
ROM^a^	*pROM* in pronation (p = 0.03)
WORKSPACE^b^	No significant differences
QOM	*D-P ratio to start* for target 1 (p = 0.003) (test 1 → test 3/4);*D-P ratio to target* for target 2 (p = 0.033) (test 1 → test 2); *Precision* on target 1 (p = 0.008) (test 1 → test 3/4); *Reaction time to target* for target 2 (p = 0.041) (not sign. post hoc);*Reaction time to target* for target 7 (p = 0.045) (not sign. post hoc);*Reaction time to start* for target 6 (p = 0.048) (test 2 → test 4)
STRENGTH	Hand opening (p = 0.013) (test 1 → test3)
RPM	Shoulder external rotation 60°/s (p = 0.001) (test 2 → test 4); Elbow flexion 60°/s (p = 0.018) (not sign. post hoc)

^a^As healthy subjects almost exclusively reached the mechanical limits ROM values correspond in most cases to the mechanical end limit.

^b^The maximal *workspace levels* and therefore the maximal *cubic volume* were reached for the evaluated subjects for all the directions (p = 1.00).

### Comparison between values of healthy subject and patients

**Fig 3:**
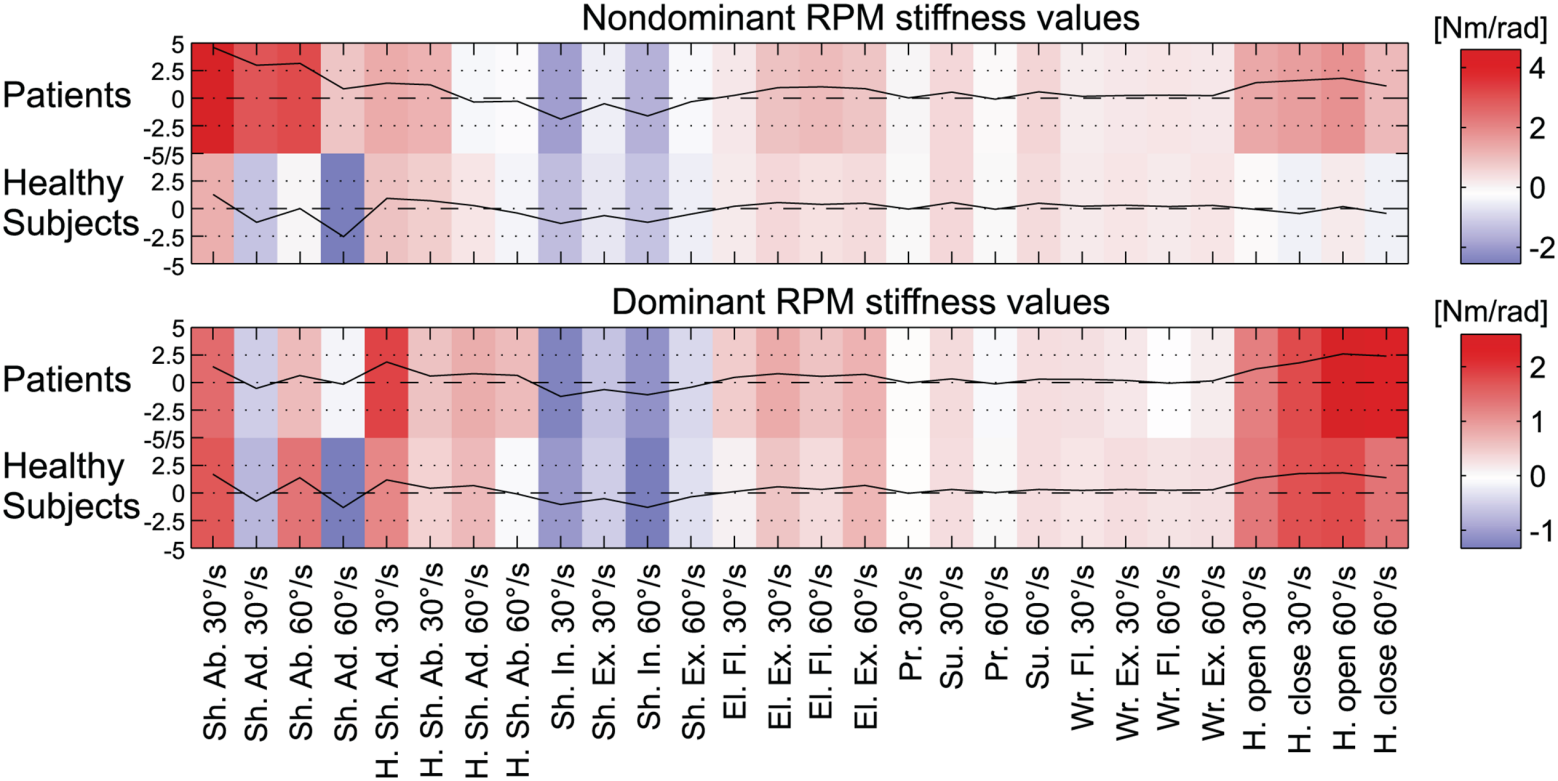
Visualization of *joint stiffness* measurements within the RPM assessment for the non-dominant and dominant arms of healthy subjects and patients. The more a joint counteracted the robot movement over the angle, the higher and more intense in color (red) the value. The color gradient ranges from minimal (blue) to maximal values (red) and is different for the non-dominant (-2 to 4) and dominant arms (-1 to 2).

The different assessment parameters were compared in the dominant (n = 5) and non-dominant (n = 4) arms. The results are listed in Table 6. The results for age- and age/gender-matched (only male patients and subjects) comparisons are similar. For better visualization of the differences between patients and healthy subjects in the RPM assessment, the average *joint stiffness* parameters for all joint movements are plotted in Fig 3.

**Table 6:** Summary of the comparison between data of patients and healthy subjects.

Assessment	Patients in comparison with healthy subjects
ROM	**Dominant arm**: *aROM*: 81%; *pROM*: 95%.
	**Non-dominant arm**: *aROM*: 67%; *pROM*: 90%.
WORKSPACE	**Dominant arm**: *cubic volume*: 79%.
	**Non-dominant arm**: *cubic volume*: 73%.
QOM	**Dominant arm**: *D-P ratio to target*: 102%; *D-P ratio to start*: 101%; *time to target*: 129%; *time to start*: 125%; *number of peaks to target*: 115%; *number of peaks to start*: 168%; *precision*: 85%.
	**Non-dominant arm**: *D-P ratio to target*: 93%; *D-P ratio to start*: 99%; *time to target*: 84%; *time to start*: 107%; *number of peaks to target*: 69%; *number of peaks to start*: 72%; *precision*: 88%.
STRENGTH	**Dominant arm**: *Joint torques* between 23% (hand opening) and 97% (elbow flexion).
	**Non-dominant arm**: *Joint torques* between 8% (hand closing) and 97% (supination).
RPM	**Dominant arm**: The difference between *joint stiffness* in the patients’ arms (mean: 0.49 Nm/rad) and the healthy subjects (mean: 0.34 Nm/rad) is 0.15 Nm/rad.
	**Non-dominant arm**: The difference between *joint stiffness* in the patients’ arms (mean: 0.72 Nm/rad) and the healthy subjects (mean: -0.08 Nm/rad) is 0.80 Nm/rad.

For *D-P ratio*, *time to start/target*, *number of peaks* and *precision* a value below 100% indicates a better performance of the patients while for *ROM*, *cubic volume* and *joint torque* a value below 100% indicates a better performance of the healthy subjects.

### Inter-rater reliability

**Fig 4:**
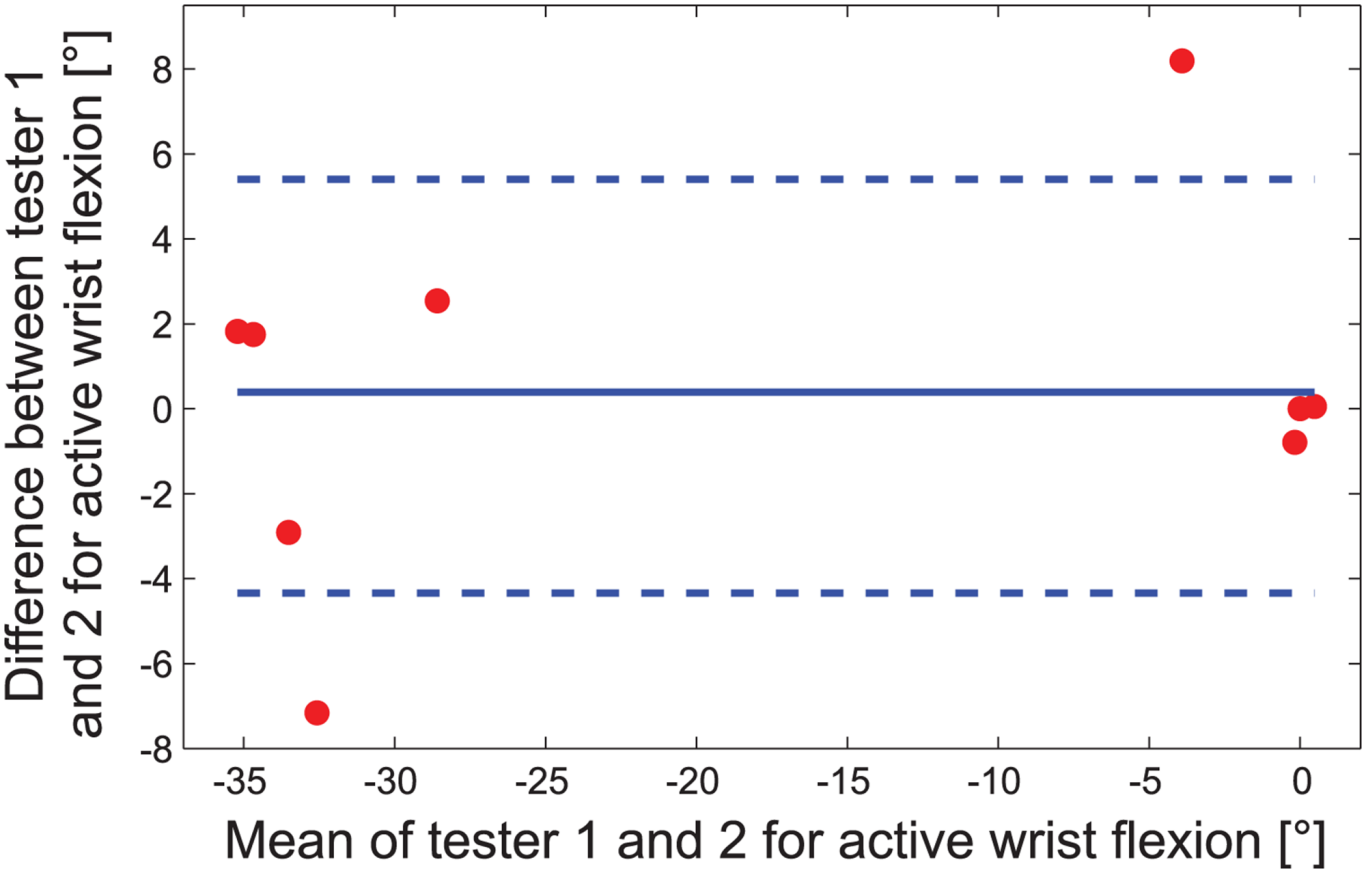
Example of an *aROM* Bland-Altman plot regarding wrist flexion for the nine patient arms. The limits of agreement (dashed lines for lower -4.3° and upper 5.4° limit) and the mean difference (solid line at 0.5°) are shown. The x-axis shows the mean values of the two measurements of tester 1 and 2 (negative values indicate flexion, positive values stand for extension), while the y-axis shows the measurement difference between tester 1 and 2.

**Fig 5:**
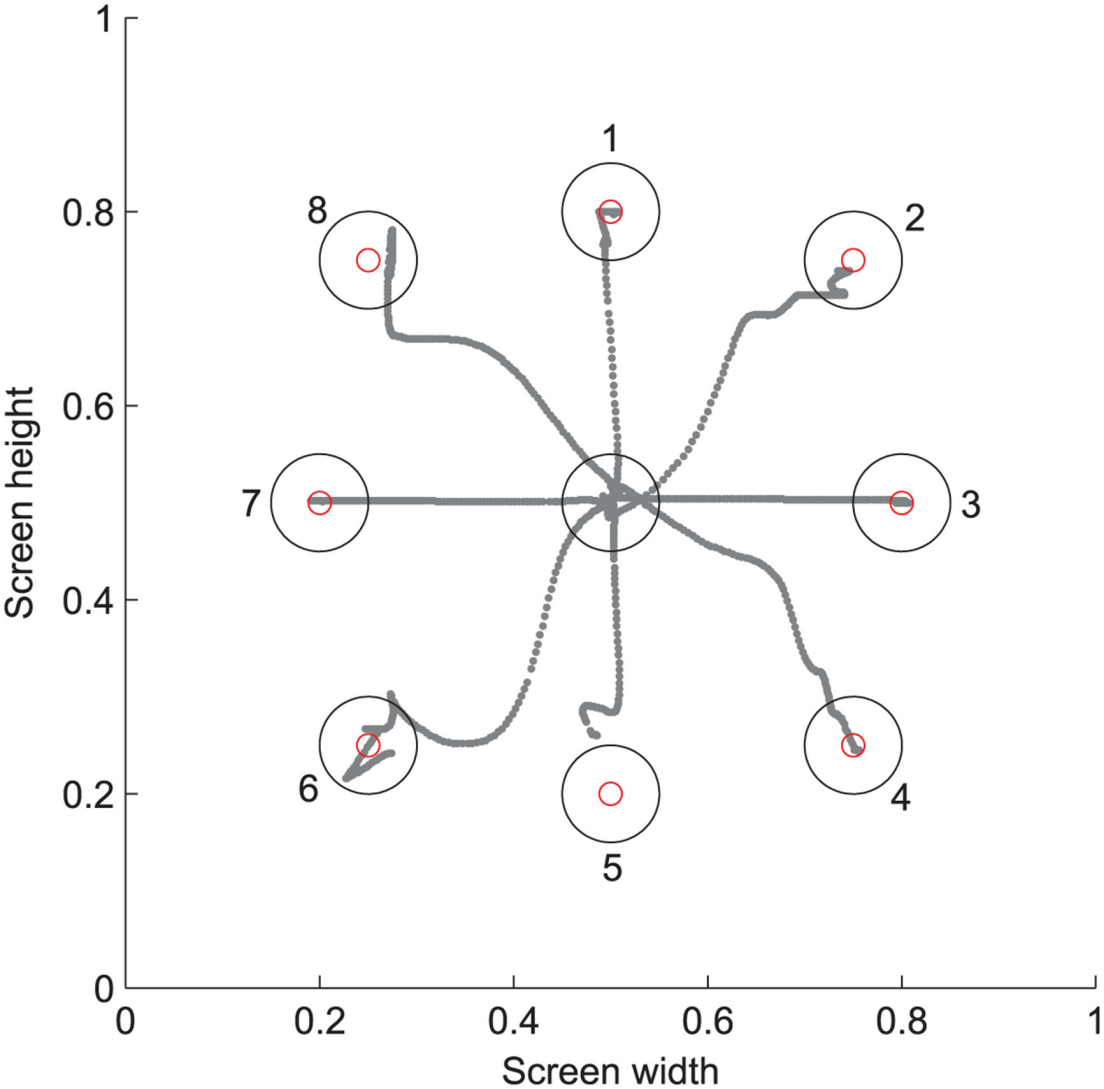
QOM: The hand paths of a patient moving to the 8 different targets.

The inter-rater reliability was analyzed using the Spearman’s rank correlation coefficient and the Bland-Altman plot. In Fig 4 an exemplary Bland-Altman plot for the wrist flexion is shown. The analysis of the WORKSPACE and QOM parameters is shown in more detail in S3 Table. The mean *cubic volume* was 107.5 dm^3^. Fig 5 is an example of a patient’s hand movements to the eight different targets in the QOM assessment package. The summarized results for the inter-rater reliability between testers 1 and 2 are summarized in Table 7.

**Table 7:** Summary for the inter-rater reliability analysis.

Assessment	Spearman correlation, ICC, Bland-Altman analysis
ROM	Spearman: *aROM*: Shoulder flexion/extension (0.95/0.85); lateral shoulder ab-/adduction (0.83/0.96); horizontal shoulder ab-/adduction (0.78/0.83); shoulder internal rotation (0.87); supination (0.92); wrist flexion/extension (0.72/0.85). *pROM*: Lateral shoulder adduction (0.98); shoulder extension (0.92); horizontal shoulder ab-/adduction (0.77/0.90); internal rotation (0.80); wrist flexion (0.67).
	Bland-Altman: *aROM*: No significant differences; *pROM*: Shoulder extension (p = 0.047), internal-/external rotation (p = 0.033/p = 0.028).
WORKSPACE	Spearman: *Workspace levels* (between 0.75* and 1.0**); *cubic volume (*0.77*).
	Bland-Altman: *Workspace levels* downwards (p = 0.035); *cubic volume* (p = 0.025).
QOM	Spearman: *Number of peaks to start*: 0.71*.
	Bland-Altman: No significant difference between testers.
STRENGTH^a^	ICC: Between 0.80** and 0.98** (except for supination *joint torque*: 0.38).
	Bland-Altman: Wrist extension (p = 0.01).
RPM	Spearman: Shoulder flexion at speed 30°/s and 60°/s; Shoulder internal rotation at 30°/s.
	Bland-Altman: Elbow flexion at 60°/s (p = 0.019).

The numbers show the significant Spearman correlation coefficients, the significant ICC values and the significant differences from 0 in the t-test for the Bland-Altman analysis.

* = p<0.05.

** = p<0.01.

^a^Data of testers 1 and 2 were normally distributed for STRENGTH.

### Construct validity

#### ROM

Spearman’s rank correlation coefficient between the clinical ROM measurement and the robot’s ROM was significant for *aROM* in lateral shoulder abduction, elbow flexion and wrist flexion/extension, and showed a moderate but not significant correlation for shoulder flexion (0.59, p = 0.06). The other correlations were not significant (p = 0.1 to p = 0.43). Regarding *pROM*, only lateral shoulder abduction and horizontal abduction were significant. The results are shown in Table 8.

**Table 8:** Summary of the results for the construct validity.

Robotic assessment	Clinical assessment	Spearman correlation
ROM	manual ROM	*aROM*: Lateral shoulder abduction (0.63, p = 0.034*), elbow flexion (0.81, p = 0.004**) and wrist flexion/extension (0.78, p = 0.007**/0.67, p = 0.035*); *pROM*: Lateral shoulder abduction (0.78, p = 0.006**) and horizontal abduction (0.64, p = 0.031*).
WORKSPACE	ARW	*Cubic volume* significantly correlates with ARW (0.69*).
	VLT, GRASSP, SCIM	*Cubic volume* correlates moderately with the test score of GRASSP (0.53), VLT (0.54) and SCIM (0.51). The *workspace levels* correlate on a 0.7* level with the VLT and GRASSP items.
QOM	VLT, GRASSP, SCIM	Particularly *D-P ratio to start* (GRASSP: -0.700*, VLT: -0.644*) and *reaction time to start* (GRASSP: -0.667*, VLT: -0.636*) show moderate correlations with the clinical scores.
STRENGTH	MMT	The correlations between STRENGTH *joint torque* and MMT range between 0.69* and 0.91** (except for shoulder extension).
RPM	MAS, MTS	No analysis due to lack of data.

The table shows the significant correlations between the robotic and clinical assessments.

* = p<0.05.

** = p<0.01.

Bland-Altman plots were generated to evaluate the degree of agreement between clinical and robotic measurements. The mean difference between the two measurement methods were significantly different regarding *aROM* for 6 out of 12 measurements (lateral shoulder adduction, shoulder extension, internal and external rotation, supination and wrist flexion) and regarding *pROM* for 10 out of 12 measurements (lateral shoulder adduction, shoulder extension, horizontal ab-/adduction, internal and external rotation, elbow flexion, pronation and wrist flexion/extension). This difference results from predominantly higher clinical ROM values compared to the ranges measured in the robot. This was also reflected in correlations between the assessment differences and the mean values in the evaluation of the Bland-Altman plots, i.e. the difference was higher, for higher mean values.

#### WORKSPACE

The arm reachable workspace (ARW) was calculated from ROM values measured with a goniometer. Spearman’s correlation coefficient between *cubic volume* and the ARW was 0.69*. The *cubic volume* was approximately half the size of the ARW.

*Cubic volume* correlates on a 0.5–0.6 level with VLT and the single prehension items of the GRASSP but not with the sensibility items of GRASSP. The correlation of *cubic volume* was 0.53 for the GRASSP, 0.54 for the VLT and 0.51 for the SCIM. The *workspace level*s in the different ARMin directions correlated on a 0.7* level with VLT and GRASSP items, and on a 0.8* level with the targets in the “up” direction, but not for the sensibility items.

#### QOM

Overall, results regarding correlations of QOM with GRASSP, VLT and SCIM were inconsistent but there was a tendency that patients with higher values in GRASSP, VLT and SCIM got better results in QOM values (Table 9). Particularly, *D-P ratio to start* and *reaction time to start* showed moderate correlations with the clinical scores.

**Table 9:** Correlations between the QOM assessment and the clinical GRASSP, VLT and SCIM scores.

QOM metric (Mean values)	GRASSP	VLT	SCIM
*D-P ratio to target*	-0.450	-0.326	-0.441
*D-P ratio to start*	-0.700*	-0.644*	-0.653*
*Precision*	0.217	0.209	0.314
*Number of peaks to target*	0.233	0.142	0.144
*Number of peaks to start*	0.000	-0.050	-0.059
*Reaction time to target*	-0.350	-0.293	-0.398
*Reaction time to start*	-0.667*	-0.636*	-0.687*
*Time to target*	0.400	0.427	0.373
*Time to start*	-0.017	0.000	0.008

Negative values result when scales have diametric changes with improvement.

* = p<0.05.

#### STRENGTH

**Fig 6:**
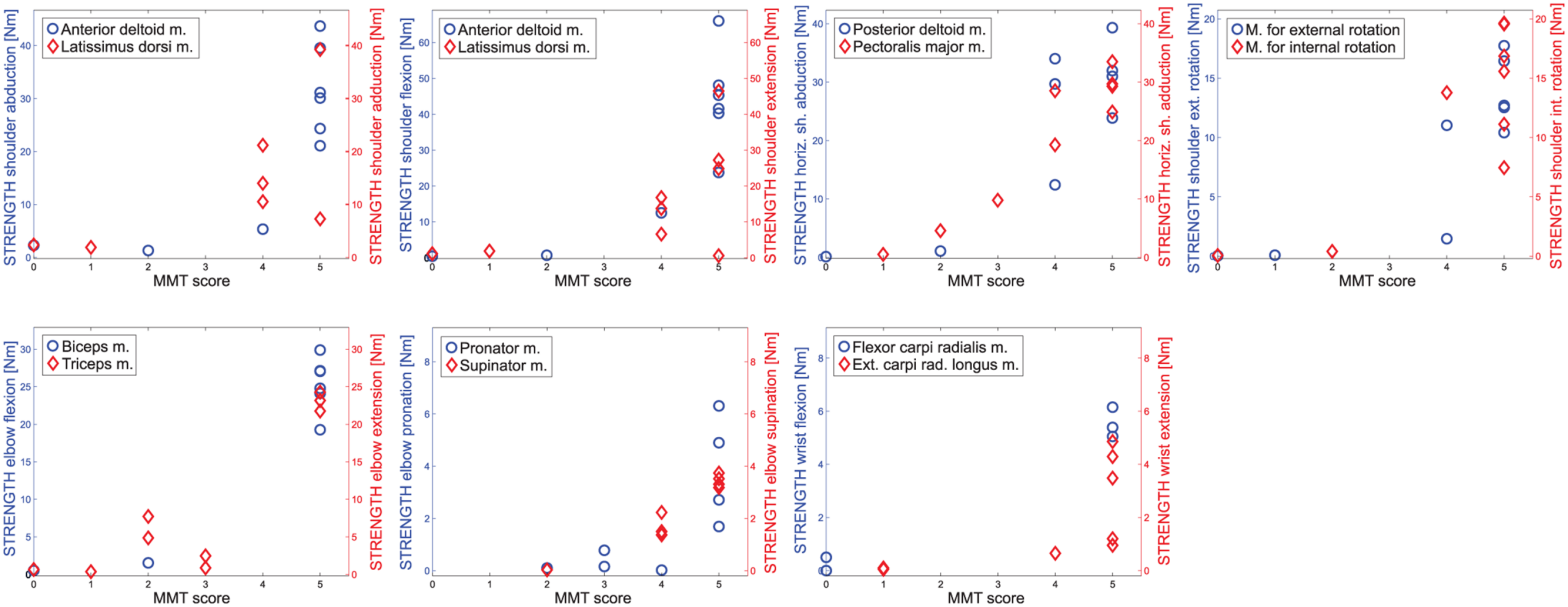
Plots of the patient *joint torques* from STRENGTH vs. MMT scores for all joints and in both directions. (x-axis = MMT score, y-axis = *joint torque* from STRENGTH).

The Spearman’s correlation coefficients between STRENGTH and the manual muscle test of corresponding joints were very high and ranged between 0.69* and 0.91**. The corresponding data for all joints is plotted in Fig 6. Only for shoulder extension there was no significant correlation (0.54, p = 0.066).

#### RPM

**Fig 7:**
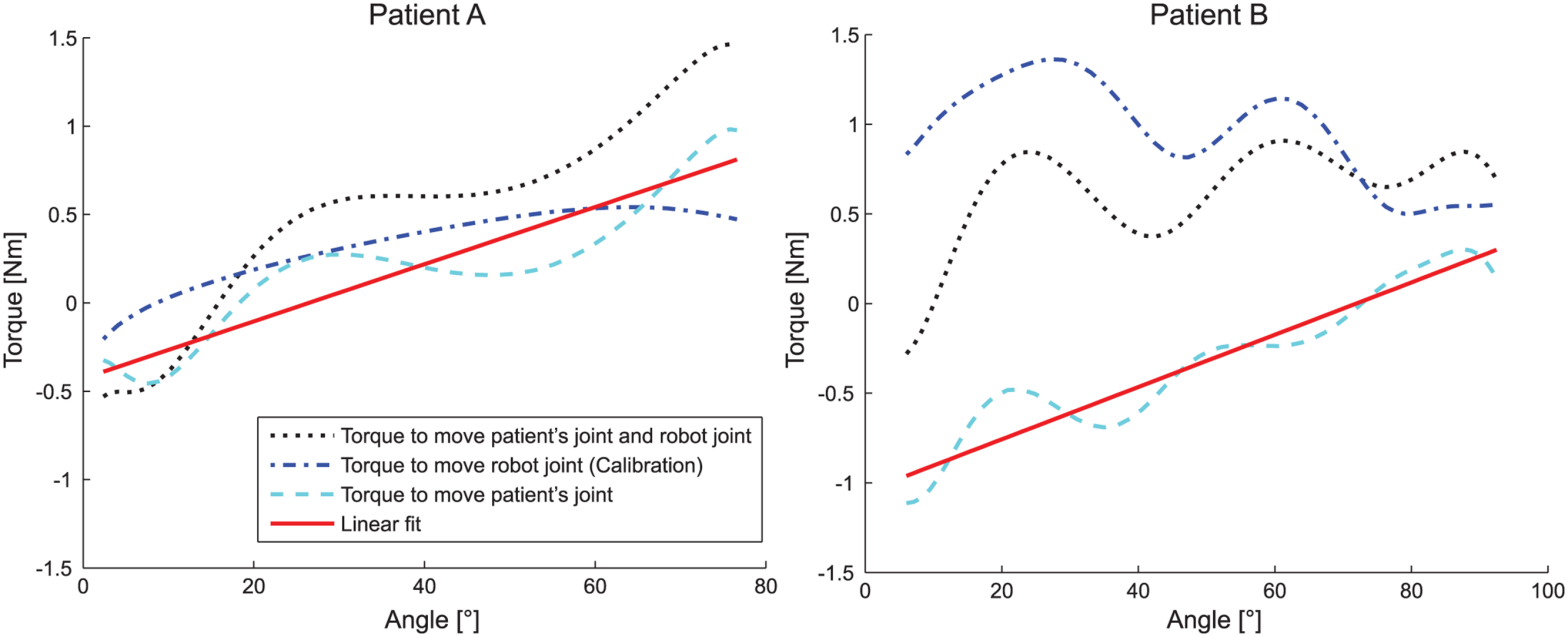
Example RPM plot for elbow flexion (60°/s) of two patients (A and B). The black dotted line shows the torque progression measured during the assessment. The blue dash-dotted line is the data from the calibration routine without the patient. The cyan dashed line is the estimated torque progression of the patient’s joint. The red line is the linear least square fit for the patient’s joint torques. The movement in the left picture was rated a 2 on the Tardieu scale. The movement on the right example was rated a 0 on the Tardieu scale. The measured stiffness by the robot assigned 0.93 Nm/rad to the left and 0.84 Nm/rad to the right movement.

Only one single patient showed a Tardieu and/or Ashworth Scale value higher than 0 (for shoulder extension, internal rotation, elbow flexion, supination and wrist extension), all other patients had no clinically detectable spasticity. Therefore, no further statistical analysis was possible to calculate the correlations. An example for RPM is shown in Fig 7. It represents both the elbow flexion data of a patient with a Tardieu score of 2 and a patient with a Tardieu score of 0.

## Discussion

Despite the small sample size, first conclusions could be drawn regarding applicability, reliability, validity and limitations of the single assessment packages. All subjects and patients were able to understand and perform the different assessment packages.

### ROM

#### Assessment evaluation

The mechanical joint limits of the robot were reached by the healthy subjects and sometimes by the patients, leading to a saturation effect of the range values. Therefore, ROM showed good intra-rater (except for *pROM* pronation) and inter-rater (except for shoulder extension and internal-/external shoulder rotation) reliabilities. The *aROM* and *pROM* values of the patients were both smaller compared to healthy subjects. Furthermore, the comparison of the *aROM* and *pROM* values with the manual ROM test was good for several joints (active lateral shoulder abduction, elbow flexion, wrist extension, shoulder flexion and passive lateral shoulder abduction). We assume that this correlation is better for severely and moderately affected patients, who do not reach the robot joint ranges. The results support our use of the ROM values as a basis for exercises or assessments (such as the RPM assessment) to define the usable and safe region.

#### Limitations

The mechanical limits of the robot are not only due to kinematic constraints but also chosen for safety reasons and, therefore, do not cover the whole range that can maximally be reached by a healthy subject (e.g. in [54]). This was reflected by the significant differences between tester 1 and 2 found in the Bland-Altman plot and the strong correlation between the differences and the mean of clinical and robotic results. As most patients in the sample got higher ROM values in the clinical measurements with the goniometer, a goniometer has to be preferred to robots (with similar ROM) for the measurement of joint range.

#### Applicability

The chosen robot postures for measuring the range were applicable for all patients. Even patients sitting in a wheelchair could be measured in the predefined positions. The discrepancy between clinical and robotic ROM in wrist extension was mainly due to an offset between the zero position of the robot (i.e. grasping the hand module leads to an initial dorsal extension) and the zero position measured by the therapist (straight hand with stretched finders and no dorsal extension).

### WORKSPACE

#### Assessment evaluation

The healthy subjects reached the maximum cubic workspace, therefore, intra-rater reliability was good regarding *cubic volume* and *workspace levels*. The comparison with the patients showed that their *cubic volume* was around 70–80% of healthy subjects (Table 6). Furthermore, a good inter-rater reliability was found for *workspace level* and *cubic volume*. However, the Bland-Altman plot showed significant differences between the two testers for *cubic volume*. A closer look at the values revealed that the volume assessed by tester 2 was always equal (4 out of 9 tests) or higher (5 out of 9 tests) as compared to tester 1. The actual source for this difference could not be identified. Possibly, the difference came from discrepancies in how the patient was motivated to reach in the different directions. The *cubic volume* correlated well with the ARW and moderately with the VLT/ GRASSP items. As WORKSPACE is reliable, it can be used for calibration of exercises (e.g. to place objects in the virtual environment, so that the patient can reach them) or as a basis for further assessments (e. g. as we did in QOM). More parameters might be extracted from this assessment to analyze the movements in more detail, such as the interjoint coordination. However, changing distances to the room walls have to be taken into account.

#### Limitations

WORKSPACE should only be used for assessment of severely affected patients, who do not reach the maximum cubic volume. The inter-rater reliability was poor in the downward direction for *workspace level*. The reason may be that downward movements were limited by the legs and depending on the robot forearm posture the robot could be moved more or less in this direction. A solution would be to fix the pro-/supination in a predefined position during this measurement such that the conditions stay the same for each patient.

Another limitation related to the clinical validity was that the absolute values of the workspace volume were much higher for the clinical assessment than in WORKSPACE, the reason being that WORKSPACE did not calculate the real workspace volume (e.g. as shown in [16]) but a cubic volume in front of the patient.

#### Applicability

No problems with the applicability of the assessment. The workspace was assessed in several steps reaching walls of discretely increasing distance. It could be simplified by continuously moving the walls away from the center position.

### QOM

#### Assessment evaluation

The QOM assessment had largely good intra-rater reliability. The only values that changed significantly over the course of the four ARMin sessions were almost exclusively due to different results for the first targets of the first session pointing to a learning effect. The comparison between patients and healthy subjects revealed that the patients performed similar or better than healthy subjects with the non-dominant arm. Currently, we have no explanation for this trend. In the dominant arm the healthy subjects gained better results (as expected, Table 6). The inter-rater analysis showed neither significant correlation between the testers (except for *number of peaks to start*) nor could differences be detected in the Bland-Altman analysis. The reason is in our opinion the small sample size. The *precision* metric showed a tendency to moderately correlate between the testers. The construct validity analysis revealed moderate but significant correlations comparing *D-P ratio to start* and *reaction time to start* with GRASSP and VLT.

#### Limitations

The fact that specifically the movements to the first target in the first test showed significant differences to later tests indicates that the first couple of movements should not be included in the assessment as it is likely influenced by learning factors or inattention in the beginning.

The results for the comparison and the inter-rater reliability showed clear differences between movements to the target and start position. This effect may be caused by a clear knowledge about the direction in which the start position will appear and, therefore, the movement could be planned before the start position showed up. For the target positions the direction was not known beforehand and the reaction and the movement may have been influenced by where the target appeared.

The results of the peak metric from the comparison between patients and healthy subjects were very ambiguous. It is known from literature that the peak metric performs fairly well for stroke patients but is insensitive, nonrobust and unsuitable for healthy subjects [25] and insensitive to brief resting periods [24]. Although our assessment was performed in the frontal and not in the transverse plane as described therein, conclusions from the *number of peaks* metric should be drawn carefully. Further metrics such as the spectral arc-length metric for smoothness [25] should be considered for evaluation in a next assessment version.

*Reaction time* would be more reliable, when measuring the time to overcome a certain distance from the real starting position, rather than measuring the time to leave a starting circle, where the real starting position inside this circle was unknown. Therefore, the distance that had to be moved until the *reaction time* was measured changed in each trial.

The location of the targets in the frontal plane without a restriction for the distance to the body may have been a source for higher variability in the assessed parameters, as the patient could chose slightly closer or more distant targets in different trials. An additional assessment package covering the quality of movement could be a tracking task of a figure e.g., a circle [10] or a Lissajous figure [55].

#### Applicability

No problems with the applicability of the assessment.

### STRENGTH

#### Assessment evaluation

The STRENGTH assessment had a good intra-rater and inter-rater reliability (except for wrist extension which showed a significant difference between the two testers in the Bland-Altman analysis). All the six valid comparisons for wrist extension showed slightly higher values in the assessments of one tester. The reason for this effect is unclear and we assume that it comes from the small number of valid samples in this joint and the normal variability in the force data.

Furthermore, the construct validity showed a very good correlation with the MMT. The STRENGTH measurement detected even small changes in torques applied which cannot be detected with the MMT score. Moreover, the MMT scores showed a clear ceiling effect (Fig 6) which was not present in the STRENGTH assessment (i.e. a maximum MMT score of 5 can still be continuously graduated with the STRENGTH measurement). The MMT is the standard assessment to measure muscle forces. Cuthbert et al [56] reported an inter-rater-reliability from 0.63 to 0.98 with very well trained testers. As the STRENGTH assessment reached ICC scores for the inter-rater reliability from 0.80** to 0.98** the robotic assessment is comparable with the MMT score. Further aspects that could potentially be investigated in a later version are endurance or fatigue.

#### Limitations

The measurement of the mid-hand closing forces may not be reproducible in other robots without knowledge of the exact design of the specific hand module.

#### Applicability

The interface was used to show the currently assessed joint and in which direction the torque has to be applied. However, difficulties in understanding the indicated direction and the correct timing were reported sporadically.

### RPM

#### Assessment evaluation

The RPM assessment aimed to measure the torque that was needed to move single joints of the arm. The stiffness portion of the arm resistance may be used as an indicator for arm stiffness or spasticity. The patients participating in this assessment study had almost no clinical relevant spasticity and, therefore, the interpretability of the results is very vague. Accordingly, the *joint stiffness* values were rather noisy with a trend for an increased resistance in patients. Mainly for shoulder ab-/adduction, elbow flexion/extension and hand opening/closing (Fig 3).

If further investigations proof the validity of RPM assessments this would be a very good alternative for clinical scales such as MTS and the MAS which are neither reliable measurements for the upper extremity [57] nor have a rational scale with an appropriate resolution to measure subtle changes in the arm stiffness.

Similar robot-assisted RPM measurements were already performed by other groups with the therapist moving the arm and measuring force and angle with sensors achieving promising results [58]. Therefore, we hold to this approach and plan more tests with patients with spasticity or biomechanical stiffness in the arm.

#### Limitations

The variation between ARMin tests on different days were high, this could be due to a real variation of the spasticity over time. Furthermore, clinical tests and the ARMin assessments were not performed on the same day or during the same time, which also influences the measured stiffness. For further studies the clinical investigation of spasticity has to be shortly before or after the RPM measurement.

Higher joint speeds would be desirable for measuring aspects of speed-dependent stiffness (as in [33] or [59]). However, the ARMin robot is not powerful enough to reach higher speeds than roughly 60°/s over a sufficient joint range (after subtracting the joint region for acceleration and deceleration of the joint).

#### Applicability

For the RPM measurements in this paper there was a calibration routine of the robot necessary in order to know the torques without patient. An accurate dynamic model of the robot would make the calibration redundant and it could be skipped.

### General remarks

In this consideration of concept study only mean values for all directional movements were used (QOM and WORKSPACE). However, in a later study when there is more patient data available, specific directions, sectors or quadrants of the workspace should be grouped for a more detailed direction-dependent analysis.

Although the presented assessment packages were implemented in the ARMin robot, the methods can be applied to other exoskeleton robots. However, for STRENGTH the actuators and the robot structure have to be strong and stiff enough to counteract the torques applied by the patient. WORKSPACE and QOM can also be applied to end-effector robots, as both assessments only need the hand position as an input.

The robot as a measuring tool may produce unwanted interaction forces with the patient. These were reduced by active compensation of both gravity and friction of the robot. However, the arm dynamics may still be influenced, e.g., by additional inertia or non-modeled gravitational and frictional effects. This makes it more difficult to compare the assessment outcomes with other platforms or measurements (e.g., a free arm movement outside the robot) or with a platform with different inertial properties. This is mainly an issue for the assessments with free end-effector movements (QOM and WORKSPACE). Furthermore, we cannot exclude that small imprecisions in the modeling of the nonlinear spring compensation may have led to a systematic bias for a certain direction in the shoulder flexion/extension joint. However, this effect could not be observed from the recorded assessment data.

Besides these modeling factors of the robot, the therapist as well has some influence on the outcome. Even though the recordings are performed by the robot (except for the pROM) the positioning, instruction and motivation of the patient—though unintended—can influence the performance during the assessment and is reflected in the observed inter-rater reliability. This influence could further be reduced by using standardized audio-instructions or self-aligning exoskeleton axes [60]. In pROM the range measurement is directly affected by the therapist’s (and also patient’s) rating of how strong and far the joint can be moved before the resistance gets too high or the movement causes pain.

The different assessments can be used independently from each other. Therefore, single assessment packages can be used to assess changes on daily basis, i.e. before or after the robotic arm therapy session. Considering the duration to perform all assessments successively, the complete assessment package may only be used for admission and discharge measurements or for outcome tests in research studies.

## Conclusions

We evaluated five robot-assisted assessment packages that are integrated into the arm therapy device ARMin and measured different aspects of the human arm motor capability in SCI patients. We found very good reliability and validity for the *joint torque* measurements despite the small number of patients. Furthermore, the measurements for the reachable cubic workspace and different quality of movement metrics showed tendencies for good intra-rater and inter-rater reliability as well as validity.

To our knowledge this is the first study integrating a comprehensive set of assessment packages in a single actuated robotic platform to measure kinematic, kinetic and timing parameters on joint and end-effector level. Furthermore, this study presents for the first time the application of an actuated arm robot for assessment of SCI patients. The actuation allows, on one hand, free arm movements (QOM and WORKSPACE) but on the other hand precise performance of desired movements (RPM) and complete resistance against the arm (STRENGTH). Moreover, the backdrivable and transparent design of the robot combined with the sensitive sensors allows for continuous and accurate measurements of arm movements and torques.

We believe that all the described methods and results can be generalized and transferred to other robotic exoskeleton platforms (e.g. the commercial version of the ARMin robot, the ArmeoPower) or other patient groups, such as stroke patients. WORKSPACE and QOM assessments can also be applied to end-effector robots, such as the ACT^3D^ [14], the GENGTLE/G [61] or the PASCAL robot [62]. By giving detailed precise instructions for conducting the assessments we offer a reproducible guidance for future assessment implementations into rehabilitation robots.

With this consideration of concept study we could show the feasibility of our assessment packages and their applicability to SCI patients. We started extending and improving the assessments based on the knowledge acquired during this study. The next generation of assessments will have to be tested with more patients to investigate the different trends found and aspects discussed in this paper and to work out the potential and possible application of the robot-assisted assessments in a clinical setting. Currently an extended set of assessments is being implemented in our new robotic platform ChARMin [63] for children suffering from cerebral palsy and other motor deficits.

We conclude that these robotic assessments are a new opportunity to assess patients and cannot only replace clinical assessments but offer a sensitive, objective and reliable measurement for more detailed insights in arm motor functions.

## Supporting Information

S1 Table ARMin postures for the different joints measured during the ROM assessment. (PDF)

S2 Table ARMin postures for the different joints measured during the STRENGTH assessment. (PDF)

S3 Table Summary of the inter-rater analysis of WORKSPACE and QOM. (PDF)

S4 Table Variability in the assessment parameters measured. For aROM, pROM, WORKSPACE, STRENGTH and RPM the mean differences are shown between the maximum and minimum values measured during the four recordings of the intra-rater reliability. For the QOM assessment the standard deviation is used as an indicator for the variability of the recorded parameters.
(PDF)
