# Supplementary material for: Robot-Assisted Arm Assessments in Spinal Cord Injured Patients: A Consideration of Concept Study
Source: PLoS One. 2015 May 21;10(5):e0126948. doi: 10.1371/journal.pone.0126948 (PMC4440615; doi:10.1371/journal.pone.0126948)
Supplement: S1 Table — (PDF) [file pone.0126948.s001.pdf]

Table S1. ARMin postures for the different joints measured during the ROM assessment.

	ROM joint measured						
	Lateral shoulder ad-/abduction	Shoulder flexion/ extension	Horizontal shoulder ad-/abduction	Shoulder internal-/external rotation	Elbow flexion/ extension	Forearm pronation/ supination	Wrist flexion/ extension
Axis 1: Lateral shoulder ad-/abduction	67 ABD	80 ABD	90 FLEX	90 FLEX	90 FLEX	50 FLEX	50 FLEX
Axis 2: Horizontal shoulder ad-/abduction	90 ABD	20 ABD	90 ABD	20 ABD	20 ABD	90 ABD	90 ABD
Axis 3: Shoulder internal-/external rotation	85 EXR	85 EXR	85 EXR	85 EXR	0	0	0
Axis 4: Elbow flexion/extension	0	0	0	90 FLEX	0	90 FLEX	90 FLEX
Axis 5: Forearm pronation/supination	0	0	0	0	85 SUP	50 SUP	50 SUP
Axis 6: Wrist flexion/extension	0	0	0	0	0	0	0

ABD=Abduction, EXR=External rotation, FLEX=Flexion, SUP=Supination.
