# Supplementary material for: Robot-Assisted Arm Assessments in Spinal Cord Injured Patients: A Consideration of Concept Study
Source: PLoS One. 2015 May 21;10(5):e0126948. doi: 10.1371/journal.pone.0126948 (PMC4440615; doi:10.1371/journal.pone.0126948)
Supplement: S3 Table — (PDF) [file pone.0126948.s003.pdf]

Table S3. Summary of the inter-rater analysis of WORKSPACE and QOM.

	Assessment parameter	Spearman's correlation coefficient	Bland-Altman t-test significance
WORKSPACE	Mean of *cubic volume*	0.77* (p=0.014)	0.025
	Workspace levels	0.75* to 1.0** (p<0.02 for all dir.)	p>0.035 (sign. for "down" dir.)
QOM	Mean *D-P ratio to target*	0.29 (p=0.535)	0.496
	Mean *D-P ratio to start*	0.48 (p=0.233)	0.298
	Mean *Precision*	0.68 (p=0.094)	0.112
	Mean *Number of peaks to target*	0.46 (p=0.294)	0.854
	Mean *Number of peaks to start*	0.71* (p=0.047)	0.923
	Mean *Time to target*	0.36 (p=0.432)	0.550
	Mean *Time to start*	0.21 (p=0.610)	0.069
	Mean *Reaction time to target*	0.10 (p=0.823)	0.887
	Mean *Reaction time to start*	0.29(p=0.493)	0.821

*= p<0.05, **= p<0.01.
