# Supplementary material for: Robot-Assisted Arm Assessments in Spinal Cord Injured Patients: A Consideration of Concept Study
Source: PLoS One. 2015 May 21;10(5):e0126948. doi: 10.1371/journal.pone.0126948 (PMC4440615; doi:10.1371/journal.pone.0126948)
Supplement: S4 Table — For aROM, pROM, WORKSPACE, STRENGTH and RPM the mean differences are shown between the maximum and minimum values measured during the four recordings of the intra-rater reliability. For the QOM assessment the standard deviation is used as an indicator for the variability of the recorded parameters. (PDF) [file pone.0126948.s004.pdf]

Table S4. Variability in the assessment parameters measured.

Mean(Range, i.e. difference between max and min value)									Mean(Standard deviation)	
	m1	m2	m3	m4	m5	m6	m7	m8		
aROM<br>[°]	47.9(4.2)<br>130.8(1.1)	46.7(3.9)<br>130.7(1.3)	-34.9(6.3)<br>123.6(2.0)	-28.6(3.5)<br>87.9(2.3)	-0.5(0.7)<br>118.9(2.1)	-88.8(2.9)<br>44.6(18.3)	-34.1(4.2)<br>37.4(4.0)	-<br>-	QOM Time<br>[ms]	1566(395)
pROM<br>[°]	50.3(7.5)<br>130.2(0.9)	47.7(3.9)<br>130.6(0.7)	-29.1(12.1)<br>121.3(2.5)	-27.5(3.7)<br>89.7(1.3)	-0.6(0.4)<br>116.7(4.1)	-89.7(1.1)<br>85.5(6.9)	-33.8(3.9)<br>36.6(3.5)	-<br>-	QOM Peak<br>[]	18.6(9.4)
STRENGTH<br>[Nm]	37.7(11.2)<br>30.6(12.2)	37.5(15.8)<br>45.5(15.4)	28.6(11.1)<br>32.5(8.0)	15.9(4.8)<br>14.8(3.9)	21.8(5.2)<br>24.3(7.3)	4.9(2.4)<br>3.1(1.7)	4.2(1.1)<br>4.6(2.1)	1.4(0.6)<br>1.5(0.6)	QOM D-P ratio<br>[]	1.19(0.18)
RPM 30°/s<br>[Nm/rad]	-1.57(3.88)<br>0.51(5.46)	-<br>-	1.03(1.23)<br>0.58(1.02)	-1.05(0.94)<br>-0.47(0.51)	0.61(0.64)<br>0.38(0.59)	0.00(0.26)<br>0.34(1.20)	0.4(0.21)<br>0.25(0.19)	1.89(1.57)<br>1.43(1.59)	QOM Reaction<br>[ms]	877(226)
RPM 60°/s<br>[Nm/rad]	-1.53(3.32)<br>0.73(4.15)	-<br>-	0.39(1.96)<br>-0.16(1.90)	-1.42(1.10)<br>-0.38(0.48)	0.64(0.51)<br>0.39(0.50)	0.09(0.24)<br>0.31(0.14)	0.47(0.26)<br>0.27(0.25)	1.67(1.38)<br>2.27(1.95)	QOM Precision<br>[m]	0.036(0.01)
WORKSPACE<br>[dm^3]	139.4(1.3)									

For aROM, pROM, WORKSPACE, STRENGTH and RPM the mean differences are shown between the maximum and minimum values measured during the four recordings of the intra-rater reliability. For the QOM assessment the standard deviation is used as an indicator for the variability of the recorded parameters. m1 = shoulder add-/abduction, m2 = shoulder extension/flexion, m3 = horizontal shoulder add-/abduction, m4 = shoulder internal/external rotation, m5 = elbow extension/flexion, m6 = elbow pro-/supination, m7 = wrist extension/flexion, m8 = hand closing/opening.
